# Value of peripheral blood eosinophil markers to predict severity of asthma

**DOI:** 10.1186/s12890-016-0271-8

**Published:** 2016-07-29

**Authors:** Julian Casciano, Jerry A. Krishnan, Mary Buatti Small, Philip O. Buck, Gokul Gopalan, Chenghui Li, Robert Kemp, Zenobia Dotiwala

**Affiliations:** 1 eMAX Health Systems, LLC, 445 Hamilton Avenue, 11th floor, White Plains, NY USA; 2 Medicine and Public Health, Division of Pulmonary, Critical Care, Sleep, and Allergy, University of Illinois at Chicago, Chicago, IL USA; 3 Teva, Frazer, PA USA; 4 University of Arkansas for Medical Sciences, Little Rock, AR USA

**Keywords:** Asthma, Blood eosinophil, Elevated eosinophil, Asthma severity, EPR guidelines

## Abstract

**Background:**

Asthma represents a significant clinical and economic burden to the US healthcare system. Along with other clinical manifestations of the disease, elevated sputum and blood eosinophil levels are observed in patients experiencing asthma exacerbations. The aim of this study was to evaluate the association between blood eosinophil levels and asthma severity defined using Expert Panel Report 3 guidelines.

**Methods:**

Patients with asthma diagnosis between 2004 and 2011 were extracted from the EMRClaims+ database (eMAX Health, White Plains, NY) containing electronic medical records linked to insurance claims for over 675,000 patients. The date of first asthma diagnosis was defined as the ‘index date’. Patients were required to have at least 1 peripheral eosinophil test (elevated defined as ≥ 400 cells/μL) in the 12 month ‘assessment’ period following the index date. We classified patients as those with mild asthma and moderate-to-severe asthma based on the pattern of medication use, as recommended by the 2007 National Institutes of Health Expert Panel Report. Logistic regression models were used to determine if patients with moderate-to-severe asthma had increased likelihood of an elevated peripheral eosinophil count, after accounting for demographics and comorbidities.

**Results:**

Among 1,144 patients with an asthma diagnosis, 60 % were classified as having moderate-to-severe asthma. Twenty four percent of patients with moderate-to-severe asthma and 19 % of patients with mild asthma had an elevated peripheral eosinophil count (*p* = 0.053). Logistic regression showed that moderate-to-severe asthma was associated with 38 % increased odds of elevated eosinophil level (OR 1.38, 95 % CI: 1.02 to 1.86, *p* = 0.04).

**Conclusion:**

Patients with moderate-severe asthma are significantly more likely to have an elevated peripheral eosinophil count than patients with mild asthma.

## Background

The American Lung Association has reported that nearly 26 million people (84.8 per 1000 people) in the US suffered from asthma in 2011, with children representing 27 % of them [1]. In terms of lifetime prevalence, asthma was reported in almost 40 million people in the US in 2011 (129 per 1000 people) [1]. Asthma attacks were recorded in 51 % of diagnosed patients, resulting in an attack rate as high 43.1 per 1000 [1].

The first Expert Panel Report (EPR) Guidelines [2] for asthma were established in 1991, focusing on patient education, environmental control to avoid asthma triggers, and assessment of asthma severity using lung function measures. Throughout the years, the EPR has been revised to reflect new research and novel treatment options; EPR-3 (2007) is the latest update [2]. Disease severity is central to EPR-3 guidance, with step-therapy recommended to address an escalating need for more drugs, at higher doses, with persistently uncontrolled disease. Asthma exacerbations undoubtedly increase the clinical and economic burden to patients and payers (emergency treatment being costlier than planned therapy), and are associated with substantial morbidity and mortality [3, 4].

Much progress has been made over the years in identifying external or environmental risk factors/triggers of asthma attacks such as allergens, pollutants, irritants, etc. [2, 5, 6]. Recently the focus has shifted to better understanding different patient phenotypes to manage risk and optimize outcomes. In order to prevent exacerbations and progression to more severe disease, it is essential to identify modifiable risk factors for asthma control specific to patient phenotypes. An emerging priority to standardize biological markers in clinical research in order to better evaluate patient outcomes with new and available therapeutic modalities is evidenced by the formation of an expert group by the National Institute of Health (NIH) to classify key biological outcome measures for federally sponsored asthma research. In the NIH Asthma Outcomes report, different biomarkers are grouped as “core”, “supplemental”, or “emerging”. Core outcome biomarkers are required to be included in NIH funded asthma clinical trials and observational studies, whereas supplemental biomarkers are optional [7]. According to the NIH Asthma Outcomes report, multi-allergen screening of IgE against different allergens is considered the only core biomarker [7]. Blood eosinophils are a type of white blood cells that are a part of immune responses and also responsible for inflammatory effects when triggered by allergens. Blood eosinophil measurement is recommended as a supplemental biomarker by the NIH asthma report [7], suggesting its optional use in NIH funded studies. Published studies evaluating its association with asthma exacerbation have reported significant association between blood eosinophil elevation and asthma exacerbations [8–10]]. Blood eosinophil measurement is inexpensive and widely collected as part of the Complete Blood Count (CBC) test.

This study aimed to correlate asthma severity (based on EPR guideline step-therapy recommendations) and eosinophil elevation. In the absence of patient reported symptom classification, we explore the use of eosinophil biomarkers to identify patients who remain at risk for chronic exacerbation despite treatment.

## Methods

### Study design and data source

We conducted a retrospective data analysis of patients with asthma diagnosis, extracted from the EMRClaims+ integrated health services database (eMAX Health, White Plains NY) of patients located in the Midwest region of the United States. The database includes administrative insurance claims from approximately 675,000 lives linked to an overlapping healthcare provider database of over 20 million electronic medical records data (EMR), including laboratory values, and provider billing files.

### Study population

The population was comprised of patients 12 years or older, who had at least two encounters (separated by at least 1 day) in the emergency room, outpatient or inpatient setting with a primary or secondary diagnosis of asthma (International Classification of Diseases-9- Clinical Modification [ICD-9-CM] code 493.xx) between January 2004 and July 2011 (“study period”). The date of the first asthma diagnosis in the study period was recorded as the index diagnosis date. The 12-month period following the index diagnosis was considered the ‘assessment period’. Patients were also required to have at least 1 eosinophil test conducted during the assessment period. To account for the masking effect of systemic steroid use on eosinophil levels, patients only having eosinophil results under 400 cells/μL and all those eosinophil tests conducted while on systemic steroids (based on days of supply plus 14 days) were excluded. This approach minimizes misclassification of patients with lower eosinophil levels due to systemic steroid use. Patients with confounding diseases states of COPD, emphysema (ICD-9-CM codes: 491.xx-492.xx, 494.xx-496.xx), Churg Strauss syndrome/Wegener’s granulomatosis (ICD-9-CM code: 446.4), eosinophilia (ICD-9-CM code: 288.3), pulmonary fibrosis (ICD-9-CM code: 516.3), allergic bronchopulmonary aspergillosis (ICD-9-CM code: 518.6), cystic and pulmonary fibrosis (ICD-9-CM code: 277.x,515), and lung cancer (ICD-9-CM code: 162.x) in the assessment period, were excluded.

### Study measures

Asthma severity was estimated using the medication use information reported in outpatient and/or inpatient pharmacy records during the assessment period using step-treatment recommendations for mild, moderate or severe disease by the EPR-3 criteria and adapted for use with retrospective data based on clinical guidance from asthma specialists (Table 1). Previous studies have defined elevated eosinophil levels at ≥ 400 cells/μL and have found very weak or no associations with severity at lower eosinophil cut-offs (≥200 cells/μL, ≥ 300 cells/μL) [8–10]. We also classified patients based on available eosinophil test results over the assessment period as “Elevated” if at least one test result during assessment period that was ≥ 400cells/μL, and ‘Normal’ if all available eosinophil test results were less than 400 cells/μL. Information on patient demographics (age, race, gender) was extracted. Comorbidities were captured and controlled for using the Charlson Comorbidity Index (CCI) score which was calculated for each patient. We also reported the top 5 comorbidities observed by the CCI.

**Table 1:** Classification of patients by asthma severity adapted from EPR-3 step-treatment recommendations

Severity	EPR-3 Step-Treatment Recommendations
Mild	• Low dose ICS, or • Cromolyn, LTRA, Nedocromil, or Theophylline
Moderate-to-severe	• Low-dose ICS + LABA OR Medium-dose ICS OR Medium-dose ICS + LABA, or • Low-dose ICS + either LTRA, Theophylline, or Zileuton, or • Medium-dose ICS + either LTRA, Theophylline, or Zileuton • High-dose ICS + LABA OR High-dose ICS + LABA + oral corticosteroid,or • High-dose ICS + LABA + _Omalizumab, or High-dose ICS + LABA + oral corticosteroid + Omalizumab, or • Omalizumab

### Data analyses

Descriptive statistics were used to compare baseline characteristics between the different severity groups. Frequency distribution of patients with elevated eosinophils versus normal eosinophils were reported and cross tabulated with asthma severity level. Chi-square tests were used to compare the proportion of patients with elevated eosinophil between those that had mild and moderate-to-severe asthma. Logistic regression was used to determine the association between asthma severity (key factor) and eosinophil elevation, adjusting for patient demographics (age, race, gender), and comorbidity burden (the top comorbidities or CCI score, separately). To further assess the appropriate cut-off level for elevated eosinophils, we utilized cut-off levels adopted in other studies and conducted sensitivity analyses by defining elevated eosinophils as the cut off at ≥ 300 cells/μL and ≥ 140 cells/μL. All data management and analyses were conducted using SAS Enterprise Guide version 4.3 (SAS Institute Inc., Cary, NC).

## Results

**Fig. 1:**
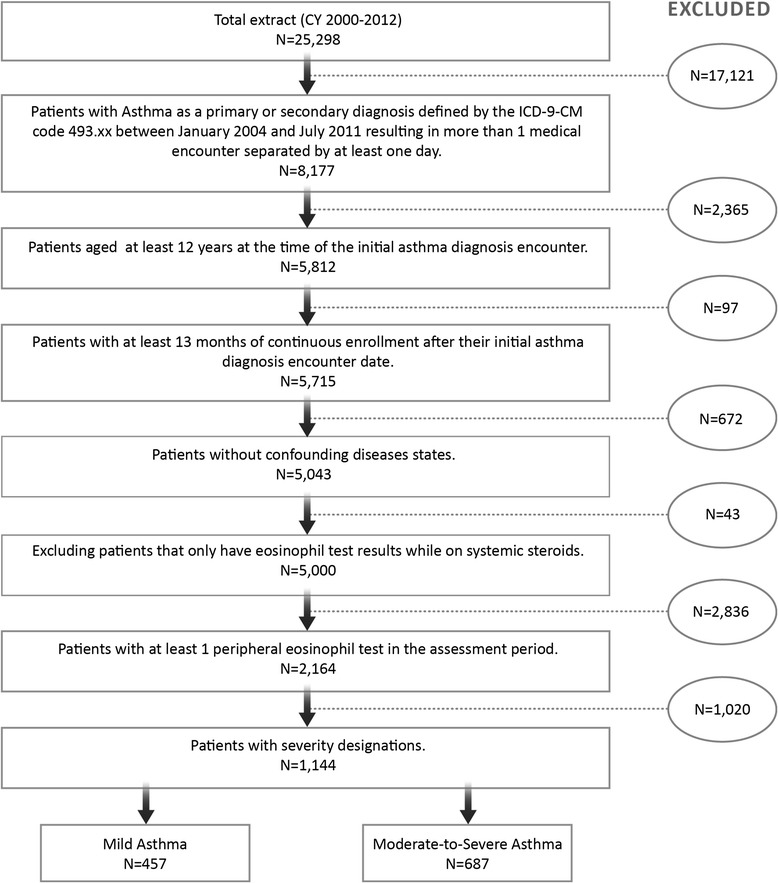
Study sample

The study identified 2,164 patients with at least one eosinophil test during the assessment period, of which 1,144 met our criteria for severity classification. Figure 1 provides a step-by-step breakdown of the study sample. Forty percent of patients were classified as having mild asthma and 60 % had moderate-to-severe asthma according to study definitions.

There was a greater proportion of women compared to men (Table 2), but the proportion was not significantly different between the severity groups (*p* = 0.116). Mean age of the overall sample was 47 years. Age distribution was significantly different between the two severity groups (*P* < 0.0001). Fifty-five percent of all asthmatics were in the 36–64 year age group; however, almost 17.5 % of patients with mild asthma were children between the ages of 12–17 years compared to 5 % in patients with moderate-to-severe asthma. Diabetes was a prominent comorbidity recorded in 14 % of asthmatic patients but the proportions of patients with diabetes were not significantly different among between the two severity groups (*p* = 0.057).

**Table 2:** Demographic and comorbidity distribution of patients by severity level

Patient characteristics	Asthma Severity		*p*-value
	Mild (*n* = 457)	Moderate-to-severe (*n* = 687)	
	N (%)	N (%)	
Gender			
Female	312 (68.27)	499 (72.63)	0.116
Race			
White	222 (48.58)	340 (49.49)	0.821
Black	59 (12.91)	81 (11.79)	
Hispanic	109 (23.85)	155 (22.56)	
Other/Unknown	67 (14.66)	111 (16.16)	
Age groups			
12–17 years	80 (17.51)	36 (5.24)	<0.0001
18–35 years	78 (17.07)	113 (16.45)	
36–64 years	232 (50.77)	401 (58.37)	
Greater than/equal to 65 years	67 (14.66)	137 (19.94)	
Top 5 Comorbidities			
Diabetes	53 (11.60)	107 (15.57)	0.057
Cancer/tumor	35 (7.66)	38 (5.33)	0.149
Congestive Heart Failure	23 (5.03)	45 (6.55)	0.288
Cerebrovascular disease	13 (2.84)	19 (2.77)	0.937
Renal disease	8 (1.75)	20 (2.91)	0.213
CCI Score Mean (SD)	1.49 (1.16)	1.52 (1.14)	0.701

**Fig. 2:**
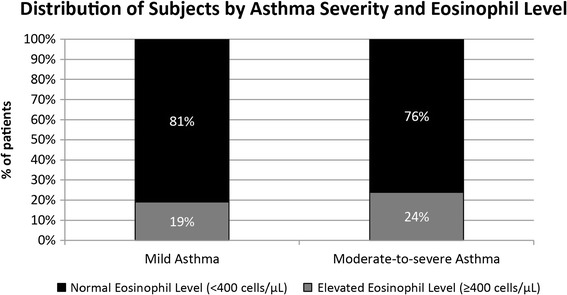
Distribution of Subjects by Asthma Severity and Eosinophil level

**Fig. 3:**
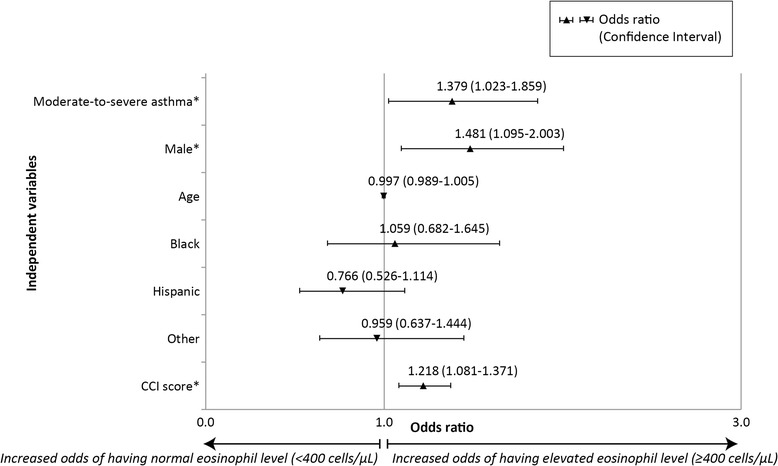
Effect of covariates on odds of having elevated Eosinophils (Moderate-to-severe asthma versus Mild). Legend: * *P* < 0.05; Reference groups: Gender: Female; Race: White; Severity: Mild asthma

Overall, 22 % of subjects had at least one elevated eosinophil level (Fig. 2). Unadjusted Chi-square analysis showed that proportions of subjects with elevated eosinophils were not significantly different between the two groups (Moderate-to-Severe 24 %, Mild 19 %, *p* = 0.053). However, logistic regression showed a 38 % increase in the odds (Odds ratio = 1.38, *p* = 0.040) of elevated eosinophils for moderate-to-severe asthma compared to mild asthma after adjusting for differences in demographic characteristics and comorbidities (Fig. 3). Males were also more likely to show elevated eosinophils.

### Sensitivity analyses

Results of sensitivity analyses conducted for elevated eosinophil level defined as ≥ 300 cells/μL showed that elevated blood eosinophils was not associated with moderate-to-severe asthma (OR: 1.07, 95 % CI: 0.83–1.38, *p* = 0.587. Similarly, elevated eosinophils at ≥ 140 cells/μL also showed no significant association with moderate-to-severe asthma (OR: 0.957, 95 % CI: 0.747–1.226, *p* = 0.728).

## Discussion

In this sample of commercially insured asthma patients aged 12 years or older, we attempted to define asthma severity by adopting recommended medications described as step therapies, by EPR 3 guidelines. This study found a clear association between asthma severity and peripheral blood eosinophils, with moderate-to-severe asthma associated with increased likelihood of eosinophil elevation. The finding is consistent with previous literature showing correlation of sputum and blood eosinophil levels in asthmatic patients. Jatakanon and colleagues [11] followed a group of patients with asthma for a period of 8 weeks, and baseline comparisons showed a significantly greater sputum eosinophil count for the group that developed exacerbations versus those that did not. The same study, through step-wise forward regression, reported that increased sputum eosinophil was associated with decreased airway function in terms of decreased forced expiratory volume (FEV) [11]. While the literature supports the association of blood eosinophil elevation with severity or functional impairment generally, identification of a clinically meaningful threshold eosinophil value seems far less clear. Here we can only offer that our observed association between severity and eosinophil at ≥ 400 cell/ μL did not hold for levels ≥ 300 cells/μL or ≥ 140 cells/μL. While this does not provide evidence for the optimal threshold, it does suggest that these lower cut-points may be of limited value in assessing patient risk. The observed association between blood eosinophil levels at ≥ 400 cell/ μL and asthma severity supports the use of routine blood eosinophil screening practices to identify the sub-segment of patients with elevated eosinophil for more targeted treatment plans.

Our findings have significant implications for the medical management of asthma. Cost and resource use rises, and quality of life decreases with asthmatic exacerbation [12]. Emergency medical treatment for exacerbation in asthma is more costly than a managed regimen [3]; moderate-to-severe asthmatics with exacerbations result in 68–88 % greater annual all-cause healthcare expenditures compared to those without exacerbation [3, 4]. In light of these facts, proactive management of asthma (especially severe asthma) has the potential to reduce the frequency of and cost incurred by these patients. The observed association of elevated blood eosinophil with moderate-to-severe in this study supports the value, among competing tests, of using blood eosinophil markers to assess patient risk in order to promote more proactive management of this patient phenotype to reduce exacerbations.

### Limitations

Requiring more than one asthma-related encounter may have resulted in over-representation of more severe patients; however, this criteria was implemented to reduce the selection of cases where a single diagnosis was assigned for suspected (but unconfirmed) asthma, a common approach when using retrospective claims data [13]. Asthma severity classification was based on medication use as opposed to observed exacerbation or impairment-based severity measures. While this classification technique may need to be validated against patient records or physician assessment, previous studies have defined asthma severity based on medication use (other than EPR-3 recommendations). Medication use serves as the best available technique in case of claims and administrative data. Lack of compliance with step-treatment recommendations may have misclassified patient asthma- severity level. We believe our classification approach is conservative, with potential misclassification which under represents the moderate-to-severe group, since poor compliance with step-treatment recommendations directionally is toward under-treatment of poorly controlled patients (as opposed to over treatment of well controlled patients). For some patients, eosinophil levels may have been defined using only one eosinophil test result over the one-year assessment period, but severity was defined based on medication use over the entire year; the temporal bias/relationship of these two variables was not assessed and should be considered as a limitation.

## Conclusion

This study highlights the association between asthma severity, defined by using EPR guidelines, and eosinophil elevation. The probability of eosinophil elevation is increased for patients with greater asthma severity. Blood eosinophil level may represent an important characteristic of disease severity as new therapeutic alternatives emerge for this patient phenotype after exhausting less aggressive medication regimes. Further research correlating blood eosinophil level with risk-based severity and control measures (such as asthma exacerbations leading to ER visits and/or hospital admissions) is warranted to externally validate the presence of our observed association. Evidence of the association between blood eosinophil levels and asthma severity underscores the need for treatment options designed for asthma patients with elevated eosinophil.

## Abbreviations

CCI, Charlson Comorbidity Index; CI, Confidence Interval; EMR, Electronic Medical Record; EOS, Eosinophil; EPR, Expert Panel Report; FEV, Forced Expiratory Volume; ICD-9-CM, International Classification of Diseases-9- Clinical Modification; ICS, Inhaled Corticosteroid; LABA, Long acting Beta agonist; NIH, National Institute of Health; OR, Odds Ratio; SAS, Statistical Analysis Software.
